# SERS Substrate Fabrication via Rapid Triboelectrification‐Driven Self‐Assembly of Close‐Packed Colloidal Monolayers

**DOI:** 10.1002/smtd.202501660

**Published:** 2026-03-01

**Authors:** Mehdi Feizpour, Ignaas S. M. Jimidar, Mitch T. J. de Waard, Gert Desmet, Heidi Ottevaere

**Affiliations:** ^1^ Department of Applied Physics and Photonics Vrije Universiteit Brussel Brussels Belgium; ^2^ Department of Chemical Engineering CHIS Vrije Universiteit Brussel Brussels Belgium; ^3^ Mesoscale Chemical Systems MESA+ Institute, University of Twente Enschede The Netherlands; ^4^ Flanders Make @ VUB‐BP&M Brussels Belgium

**Keywords:** analytical methods, photonics, SERS planar substrates, surface‐enhanced Raman spectroscopy, triboelectrification‐driven colloidal assembly

## Abstract

Surface‐enhanced Raman spectroscopy (SERS) amplifies Raman signals on nanostructured metallic surfaces, enabling the detection of trace analytes. There has been a spur on the precise fabrication of conventional planar SERS substrates with high performance and reproducibility. Common methods, such as ion beam and colloidal lithography, provide high‐quality substrates but are often limited by high costs, complex processes, and challenges associated with mass manufacturing. Triboelectrification‐driven self‐assembly of dry colloidal powder offers a promising dry approach to attain ordered monolayer colloidal particles in <20 s rapidly. Here, we use this approach to produce high‐performance SERS substrates with reproducible signals. By optimizing particle size and gold coating thickness, we found that self‐assembled 500 nm particles with a 50 and 65 nm Au layer achieved a maximum enhancement factor (EF) of 1 × 10, and limit of detection (LOD) of 33–36 nM, in our initial characterization study without coordinate translation. Compared with commercial substrates (Silmeco; Hamamatsu) under matched conditions, this corresponds to 20–24 × lower LOD (33–36 nM vs. 680–800 nM) and 10–100 × higher maximum EF. These results highlight triboelectrification's ability to efficiently and cost‐effectively produce homogeneous monolayers, offering a promising alternative to more complex or expensive methods and unlocking the opportunity for large‐scale SERS substrate production with biosensing, diagnostics, and chemical detection applications.

## Graphical abstract

Triboelectrification‐driven self‐assembly of dry colloidal powder offers a promising dry approach to rapidly (<20 s) attain ordered monolayer colloidal particles for surface‐enhanced Raman spectroscopy applications.

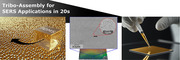

## 1 Introduction

Raman scattering is a type of inelastic light scattering used for identifying molecular structures [1]. It involves an energy shift of laser photons, observable in a Raman spectrum, which reveals the chemical composition of samples for identification and quantification [2, 3, 4]. However, Raman scattering is inherently weak, resulting in low sensitivity and long measurement times. Surface‐Enhanced Raman Spectroscopy (SERS) can amplify Raman signals by several orders of magnitude when molecules are adsorbed on their nanostructured metallic surfaces [5]. This amplification enables the detection of analytes at trace levels [6], often down to single‐molecule sensitivity [7], making SERS an essential tool for fields like biosensing [8, 9, 10], environmental pollutant detection [11] and even forensic analysis [12]. For practical applications, SERS substrates can be broadly categorized into two types: suspension‐based, e.g., colloidal nanoparticle suspensions inside microfluidic channels, and planar substrates, such as nanoparticles or nanostructures fixed onto solid surfaces. Compared to nanoparticle suspensions, planar substrates are superior in signal reproducibility, spatial signal heterogeneity, and ease of handling, which is particularly critical for industrial and large‐scale applications [13]. However, the challenge lies in developing planar substrates that are both uniform and cost‐effective, pushing researchers to explore various innovative fabrication techniques to improve the performance and accessibility of SERS [13, 14, 15].

High‐quality SERS substrate fabrication is crucial for obtaining reliable, sensitive, and reproducible signals. Among the most commonly used methods are expensive lithography‐based techniques, such as ion beam lithography, that allow for the fabrication of intricate nanostructures [16]. Ion and electron beam lithography, followed by etching, can produce substrates with exceptional control over the nanostructure geometry [17]. However, focused ion beam (FIB) lithography can directly write structures without etching. Raja et al. used FIB to directly mill sub‐micron grooves into aluminum films grown on sapphire using molecular‐beam epitaxy under ultrahigh vacuum at low temperatures [18]. The limitations of these techniques include high cost, low throughput, and the need for complex, time‐consuming processes, making them less practical for large‐scale or affordable applications. Colloidal lithography [19] is another widely explored method where colloidal particles are first self‐assembled into a close‐packed monolayer on the substrate, and then metal is deposited over the assembly. After removing the colloidal template, the remaining metal forms nanostructures with well‐defined gaps. Cai et al. created a TiO_2_/SiO_2_ inverse opaline structure using polystyrene beads of different sizes assembled on glass through a tetraethyl orthosilicate (TEOS) or titanium(IV)‐bis‐lactato‐bisammonium dihydroxide (TiBALDH) suspension [20]. Colloidal lithography offers much greater simplicity, controllability, and cost‐effectiveness than ion or electron beam lithography. Nevertheless, it can be challenging to control the size distribution and arrangement of the colloidal particles, as well as manage the complexity and reproducibility of the process. These factors often lead to variability in the substrate's performance. Soft lithography techniques, such as microcontact printing and replica molding, have also been explored as mass‐manufacturable and low‐cost methods for fabricating SERS substrates. The former involves using an elastomeric stamp to transfer nanoparticle inks or metallic nanostructures onto a substrate, while replica molding uses a patterned mold to cast nanoscale features into a substrate material. Mukherjee et al. used Polydimethylsiloxane (PDMS) stamps to transfer thin strips of air‐dried ZnO/rGO and ZnO/Ag/rGO nanocomposite films onto a new substrate, which they investigated as potential bacterial anti‐adhesion surfaces [21]. The mentioned techniques offer large‐scale fabrication and are well‐suited for producing large‐area substrates, but achieving defect‐free patterns remains a crucial challenge. Overall, while a wide variety of fabrication techniques have been developed for SERS substrate production [1], most methods face trade‐offs among precision, cost, mass manufacturability, and reproducibility. These limitations create a significant gap in the search for a fabrication method that can produce high‐performance, homogeneous SERS substrates cost‐effectively and rapidly. This gap underscores the need for innovative approaches to address these challenges while maintaining the necessary performance characteristics for SERS applications.

Self‐assembly techniques have emerged as a promising solution to these challenges, offering a quick and inexpensive route to fabricating nanostructured surfaces comprising close‐packed colloidal monolayers [7, 22, 23, 24], e.g., silica, polystyrene metal particles, in which the gaps between the particles serve as SERS hotspots [25, 26, 27]. These substrates are made SERS active by evaporating a metal layer on these colloidal monolayers [28]. Self‐assembly relies on the natural tendency of nanoparticles to organize themselves into ordered structures, driven by forces such as van der Waals interactions, capillary forces, and electrostatic forces. Lee et al. spin‐coated silicon substrates with a polystyrene suspension, allowed the particles to self‐assemble upon air‐drying, coated them with silver, and decorated them with gold nanoparticles [29]. They reported a 10 pM level sensitivity for glutathione, an antioxidant. Instead of spin‐coating, drop‐casting is also frequently used to attain close‐packed monolayers.

On the other hand, Song et al. achieved large‐area self‐assembled monolayers of polystyrene particles with single and binary sizes at air/water interface via ultrasonic spray [27]. They used these monolayers to create hexagonally packed nanopillars in silicon wafers and showed a 10–14 M detection limit for explosive 2,4,6‐trinitrotoluene (TNT). However, wet self‐assembly methods still face issues related to a lack of versatility and mass‐manufacturability in terms of particle and solvent properties (size and density), nanoparticle aggregation, incomplete surface coverage, and the need for post‐assembly modifications.

A more recent advancement in the colloidal assembly field is the development of a promising dry assembly method in terms of rapid assembly times, versatility and reproducibility. Additionally, solvent‐free assembly methods contribute to the sustainability of fabrication processes by reducing the use of solvents [23, 30, 31, 32, 33]. One approach includes the triboelectrification‐driven rubbing assembly [34, 35], where nanospheres are induced to form ordered monolayers through electrostatic interactions and mechanical deformations between particles and surface [30]. This technique harnesses the charge generated by friction between materials–like static electricity–to drive the assembly of dry powder into highly ordered arrays after rubbing them on the substrate in less than 20 seconds [30, 35]. These monolayers can be assembled on robust fluorocarbon‐coated substrates, which is a viable alternative to traditional expensive SERS fabrication techniques, with potential applications extending to biosensors, diagnostics, and chemical detection.

To meet the need for innovative solutions to fabricate cost‐effective and reproducible SERS substrates on a large scale, the present study capitalizes on the unique advantages of tribocharging‐driven rubbing assembly of close‐packed colloidal monolayers [34, 35], combined with subsequent metal coating, to fabricate high‐performance SERS substrates. Close‐packed monolayers of silica colloids with a size of 300, 400, and 500 nm were assembled on fluorocarbon‐coated silicon substrates and subsequently covered with a gold layer of varying thickness. We performed SERS characterization measurements to evaluate key performance metrics using the SERS performance analyte, trans‐1,2‐bi‐(4‐pyridyl) ethylene (BPE), including the enhancement factor (EF), the limit of detection (LOD) and spatial signal heterogeneity in terms of coefficient of variation (CV) of the fabricated substrates. We optimized the gold layer thickness for improved SERS performance. Additionally, we correlated the SERS performance to the morphology of the assembly using coordinate‐translated measurements. We tested analyte concentrations ranging from 0.2 to 15 µm to assess the substrate's performance at various detection limits. The performance metrics of the proposed studies are compared to those of commercially available, state‐of‐the‐art substrates fabricated using maskless reactive ion etching on silicon or nanoimprint technology to create highly ordered nanopillar arrays in silicon.

## 2 Results and Discussion

Key parameters for creating a SERS substrate covered with close‐packed monolayers of self‐assembled particles include the availability of monodisperse particles, metal coating, substrate material, and the size and material of the particles. Since suppressing the background signal from the substrate and particle material depends on the coverage and thickness of the metal coating, we chose a silicon substrate with silica particles. Unlike polymers, silicon and silica only have a few sharp peaks that do not interfere with the characteristic peaks of BPE. These combinations of particle size and coating thickness are known from previous research to exhibit strong absorption in the near‐infrared region of the optical spectrum [36]. We expect that smaller particles will generate a higher lateral hotspot density, while the volume of the interstitial space between larger particles increases proportionally with particle size. Thinner coatings create tighter gaps, i.e., higher‐quality hotspots; however, their plasmonic effect may be smaller. Thicker coatings can produce more uniform signals, albeit at the cost of wider gaps, i.e., lower‐quality hotspots.

### 2.1 Homogeneity of the Fabricated SERS Nanostructures

Park et al. previously introduced the dry rubbing assembly method, in which they assembled close‐packed monolayers comprising polystyrene colloids on polydimethylsiloxane (PDMS) substrates [31]. However, PDMS surfaces are inadequate for SERS, as PDMS signals interfere with characteristic peaks, and PDMS shrinks and becomes damaged when a metal layer is evaporated onto the colloidal monolayers present on PDMS to make it SERS‐active.

As such, we employ a different approach recently proposed by Jimidar and co‐workers to attain close‐packed monolayers on fluorocarbon‐coated silicon substrates based on the concept of tribocharging and contact mechanics forces [30, 34, 35]. These substrates are robust when a gold metal layer is deposited and do not interfere with the SERS signals. It has previously been shown by Jimidar et al. [34] that the particles charge positively and the fluorocarbon‐layer negatively [30, 35]. The interested reader is referred to other reported studies [35, 37] that elaborate on the rubbing assembly in detail.

**Figure 1:**
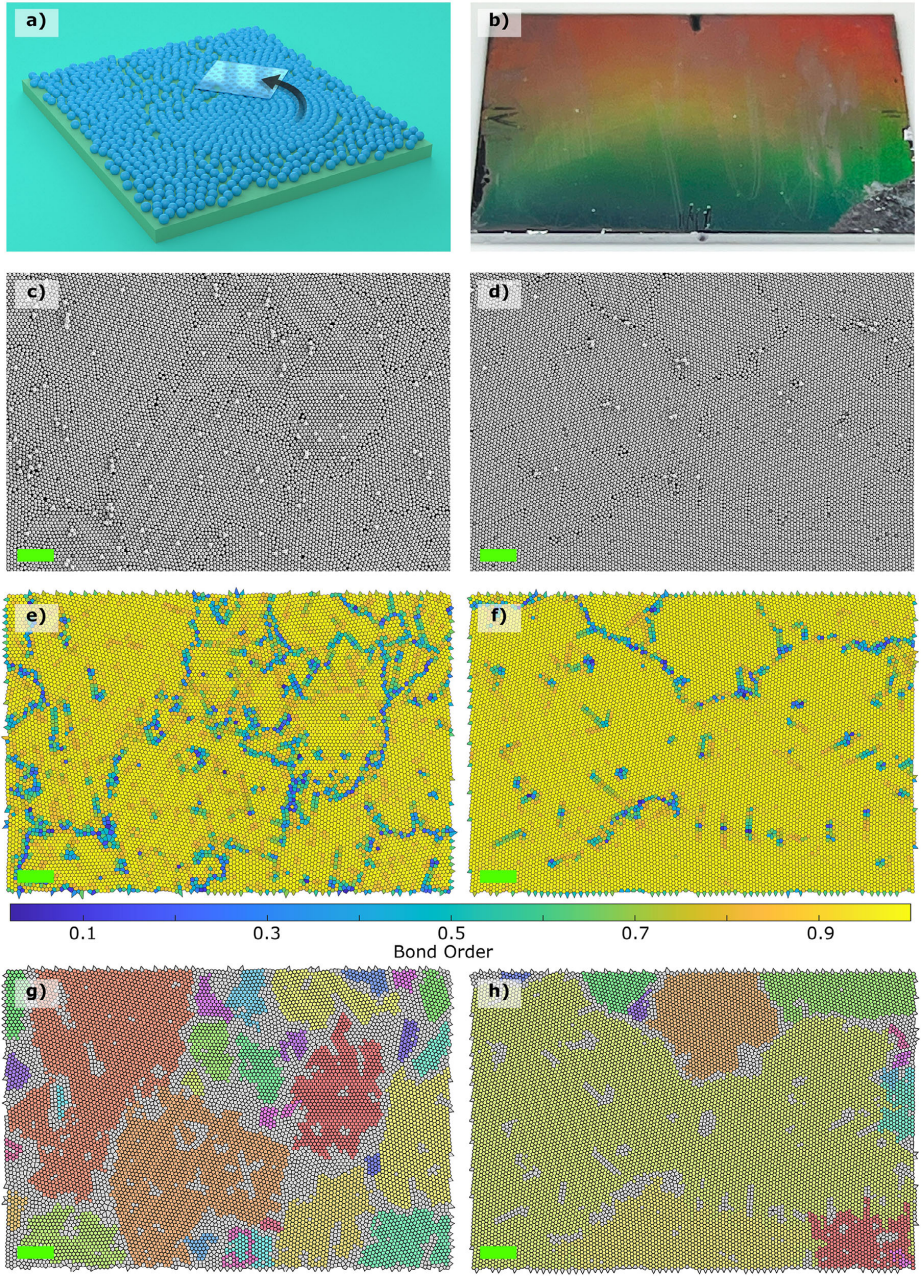
(a) Schematic illustration of the manual rubbing technique to assemble dry powder into closely packed crystal (HCP) structures on substrates using a PDMS (rubber) stamp. (b) White light illumination showing ordered diffraction patterns on the 500 nm silica colloidal monolayer assembled on fluorocarbon‐coated silicon substrate. SEM image of assembled close‐packed monolayers comprising 500 nm (c) silica and (d) PMMA colloids. (e,f) The corresponding Voronoi diagrams of the SEM images in (c) and (d) colored according to each cell's $\Psi_{6}$‐value. (g,h) The grains of the assembled monolayer crystals corresponding to (c) and (d). scale bar: 5 μm.

Figure 1a shows that the dry colloidal powder is manually rubbed on fluorocarbon‐coated silicon substrates using a PDMS sheet. In <20 s, close‐packed monolayers can be attained. We assembled monolayers of monodisperse 300, 400, and 500 nm silica and 500 nm poly(methyl methacrylate) (PMMA) colloids. It should be remarked that the silica colloidal particles needed to be assembled under controlled humidity conditions inside a glovebox (RH = 0%), whereas the PMMA particles were assembled under ambient lab conditions. This is attributed to strong capillary interactions between the silica particles, resulting in aggregated structures that hinder the monolayer assembly of silica under laboratory conditions. These capillary interactions are reduced inside the glovebox, which disrupts the aggregates and allows for single silica colloids to be assembled in a close‐packed conformation. As the more hydrophobic PMMA colloids encompass relatively weak cohesive interactions, they could be assembled under normal conditions.

Figure 1b shows a macroscopic image of the substrate under white light illumination, displaying vivid iridescent colours that highlight the large structural order of the attained silica monolayers on a 20 × 20  mm^2^ sample.

Figure 1c,d represent SEM images showing the assembled monolayers of 500 nm silica and PMMA particles, respectively. The close‐packed monolayer can be readily observed, including local defects such as particles scattered on top of the monolayer and vacancies within it. To quantify the structural order of the assembled monolayer, we use the Voronoi tessellation approach and calculate the sixfold symmetry local bond order parameter $\Psi_{6}$ [30, 35, 38]. The Voronoi cells have a hexagonal cell with $\Psi_{6}=1$ for a perfect HCP crystal. From the Voronoi tessellation diagrams (Figure 1e,f), it is understood that, due to the weaker interactions among the PMMA particles, a better and more uniform close‐packed ordered monolayer is attained with the PMMA colloids ($\Psi_{6}=0.9\pm 0.1$) than using the silica colloids ($\Psi_{6}=0.9\pm 0.2$). We assessed the quality of the assembled monolayers comprising 500 nm colloids for five substrates (four images per substrate) and found the variation in $\Psi_{6}<3$ %. In conjunction with SEM images, the Voronoi diagrams show that structural disorder can be ascribed to particles scattered on top and grain boundaries between adjacent HCP crystals, reflecting that the average $\Psi_{6}<1$.

It can also be inferred from Figure 1g,h that large HCP crystal domains (grains) can be assembled with the PMMA colloids, i.e., enhanced structural order is attained with PMMA. In particular, for the silica case, 32 individual grains are identified (Figure 1g), whereas only 14 grains are counted for the PMMA colloids (Figure 1h). However, 86% of the PMMA colloids belong to an assembled grain, while this marks up only 68% for the silica ones. These results elucidate that long‐range structural order, i.e., fewer vacancies and grain boundaries, can be achieved with the PMMA colloids than with the silica particles. The larger number of grains in the case of the silica particles signifies local order and indicates the presence of defects, such as vacancies and grain boundaries. These identified grains indicate that many nucleation sites exist to initiate the process of HCP crystal formation. However, strong cohesive interactions [23] limit large‐scale structural order, as the assembled grains on the fluorocarbon‐coated substrates cannot grow into a single‐crystal domain due to local defects. These defects are also present in wet assembly approaches. The influence of such grain boundaries, vacancies, and secondary excess particles on the SERS performances will be elaborated in Section 2.3. The interested reader is kindly referred to for the elaborate procedure for grain identification [30].

Although PMMA beads are promising colloids for forming large‐scale, structurally ordered monolayers, their interference with the SERS signal, even after gold coating, makes them an infeasible route for SERS substrates. Figure S1 highlights the differences in SERS spectra between PMMA and silica particles, showing that PMMA exhibits a higher background signal and standard deviation compared to silica. As such, the remainder of the article focuses solely on silica colloids.

### 2.2 Simulation of Particle Size and Coating Thickness

**Figure 2:**
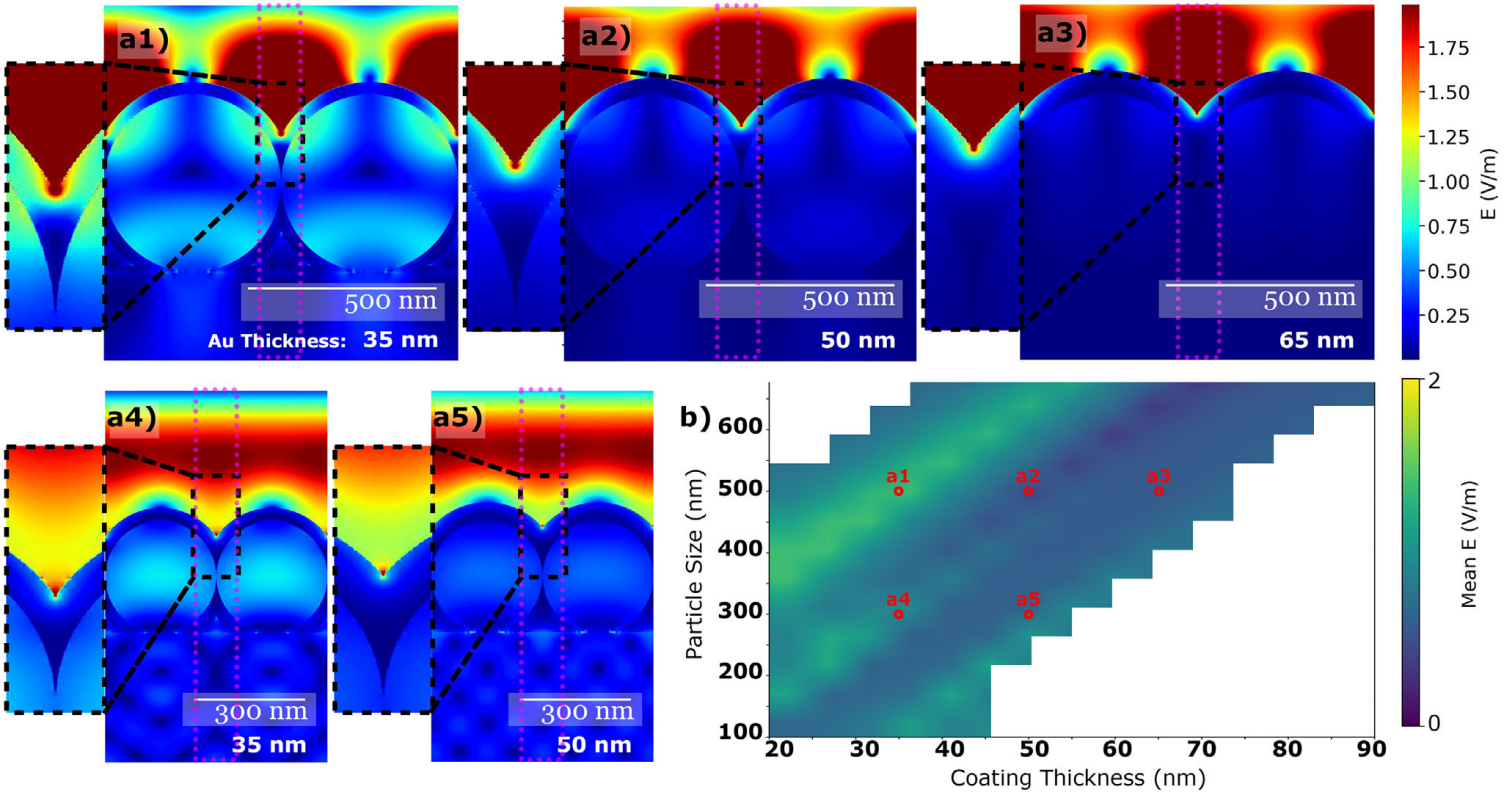
Lumerical FDTD simulation of 500 nm silica doublet coated with 35 nm (a1), 50 nm (a2), and 65 nm (a3) gold coating, as well as 300 nm doublet with 35 nm (a4) and 500 nm (a5) gold coating. Panels (a1–a5) show spatial maps of the field, E (V/m), in a vertical cross‐section through the dimer. (b) Heatmap of the spatially averaged field, computed within the magenta window for each particle‐size and coating‐thickness combination. The 0.1 µm × 1 µm magenta dotted rectangles mark the fixed averaging window used for panel (b) and are shown in all sub‐panels (a1–a5). The source amplitude was set to $E_{0}=1$, so values are numerically identical to the dimensionless $E/E_{0}$. Parameter combinations that exhibit non‐physical stair‐casing artefacts or lie outside the experimental design space, e.g., 100 nm particles with 90 nm coating, are omitted and shown as white cells. Markers in (b) indicate the particle‐size and coating‐thickness combinations corresponding to panels (a1–a5).

To explore the geometrical and optical implications of coating the particle assembly, we simulated a silica doublet on a silicon substrate with varying gold coating thicknesses, assuming uniform coverage. The 2D electromagnetic response of this configuration was computed using a finite‐difference time‐domain (FDTD) method (Lumerical FDTD, Ansys). A doublet configuration was chosen to represent the SERS substrate, as it captures essential inter‐particle gaps and near‐field interactions critical for hotspot generation. Imitating the assembly in Figure 1, the silica spheres are in contact prior to coating; directional Au deposition therefore produces metal caps separated by a shadow‐defined nanoslit at the inter‐sphere cusp. The coating was modeled as a circular shell fully covering the particles. In Figure 2 a1–a5 we plot the magnitude of the electric field E (V/m). Figure 2b reports the spatial average of this field within a fixed window (identical size and position across all cases); after artifact screening, entries impacted by mesh stair‐casing or outside the experimentally relevant coating thickness and particle size combinations, e.g., coating thickness ≈ particle size, range are blanked. Figure 2 a1–a3 show an increasing gold coating thickness on 500 nm particles. As expected, thicker coatings reflect more light, minimizing the transmission through the particles. This increases the number of photons available on the surface of the assembly to interact with analytes and scatter. However, with a thicker coating, the gap between the particles becomes shallower, decreasing the field localization, i.e., the hotspot's strength. The high field observed above the particles arises from interference between the incident and reflected fields; this behavior differs from the classic isolated dimer cases commonly shown in the literature [39] and is expected due to the different geometrical configuration.

A similar trade‐off is observed when comparing Figure 2 a1 (500 nm particles) and Figure 2 a4 (300 nm particles). With the same coating thickness, the smaller gap between the 300 nm particles fills more quickly. This shows that each particle size has an optimal coating thickness–beyond that, thicker coatings diminish field localization between the particles. Figure 2b summarizes the field localization for different particle sizes and coating thickness configurations by means of averaging the field in a window between the particles. The yellow regions, representing the maxima, clearly highlight the mentioned trade‐off. To provide a SERS‐relevant summary of these near‐field results, the simulated fields were additionally expressed in terms of an electromagnetic enhancement factor using the standard $E^{4}$ approximation, and the resulting simulated EF statistics are compiled in Table S1 shown in the Electronic Supporting Information (ESI).

**Figure 3:**
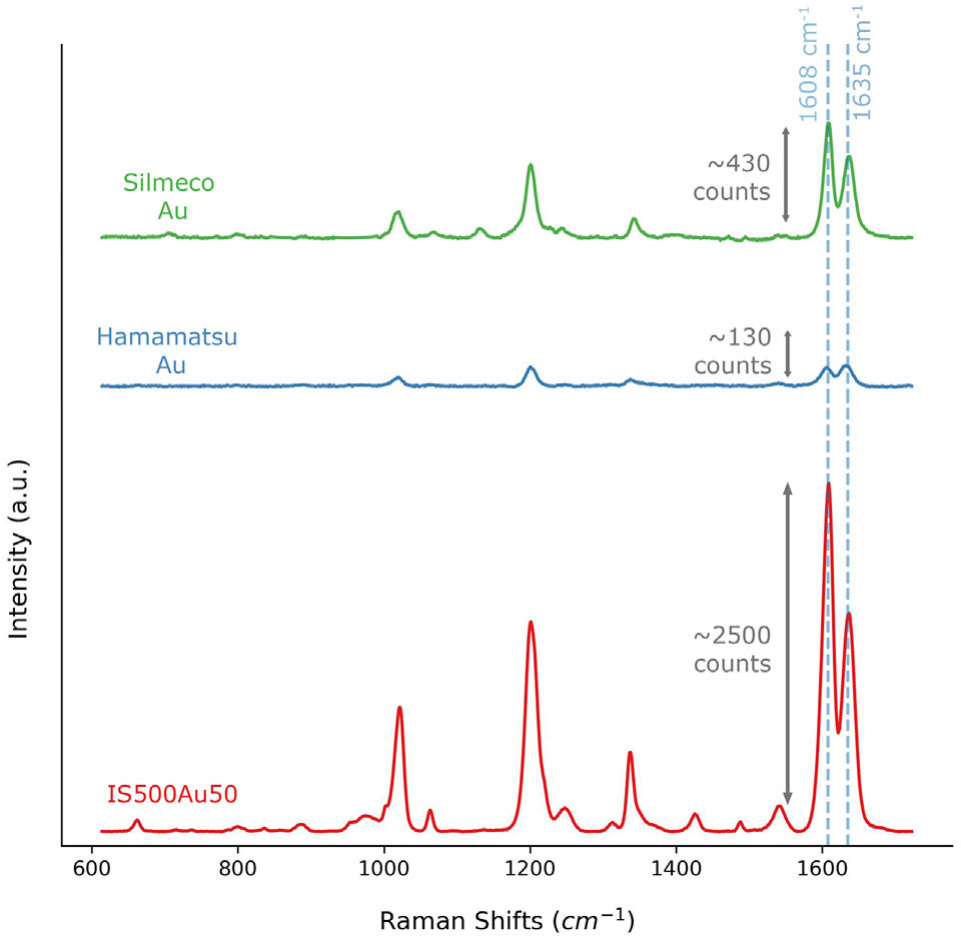
A comparison of the SERS spectra for the IS500Au50 substrate against commercial substrates. These results are based on a 1 µM BPE deposition.

Figure 2b does not point to a single best geometry. Instead, it shows a broad band of strong performance: the window‐averaged field traces a broad diagonal ridge across particle sizes of 300–500 nm and Au coatings of 35–65 nm. As the gold layer gets thicker, the averaged field steadily rises and then levels into a shallow plateau for larger thicknesses. To determine the actual optimal particle size and coating thickness for SERS performance, one needs to conduct experimental investigations. To evaluate how gold coating thickness affects SERS performance, monolayer substrates of 300 and 500 nm particles were coated with gold layers of 20, 35, 50, and 65 nm and mapped in visually selected, homogeneous areas. The results are elaborated in Section S1. As shown in Figure S2 and Table S2, EF increased with coating thickness, peaking at an optimal thickness of 35–50 nm for 300 nm particles and 50 nm for 500 nm particles. The results in Table S2 show that the best performance is obtained for the 500 nm colloids covered with 50 and 65 nm of gold, contrasting the simulation results. Note that the simulations were performed under idealized conditions–a dimer under plane‐wave illumination with perfectly absorbing top and bottom boundaries and laterally periodic (infinitely repeating) side boundaries. In practice, coating and assembly imperfections and the Gaussian beam profile deviate from these assumptions, which we identify as the primary sources of the simulation‐experiment discrepancy. The IS500Au50 sample (500 nm particles, 50 nm gold) reached a maximum EF of 1 × 10^8^ at the 1605 ${\mathrm{cm}}^{-1}$ peak of BPE, achieving a tenfold increase over Silmeco Au and nearly 100‐fold over Hamamatsu Au substrates. SERS spectra (Figure 3) reveal that IS500Au50's 1608 ${\mathrm{cm}}^{-1}$ peak is six times stronger than Silmeco's and 19 times stronger than Hamamatsu's. The coefficient of variation (CV), i.e., spatial signal heterogeneity, detailed in Figure S2c, was lower for 500 nm particles, with IS500Au50 achieving the lowest CV (9%).

### 2.3 Effect of Particle Distribution on SERS Performance

**Figure 4:**
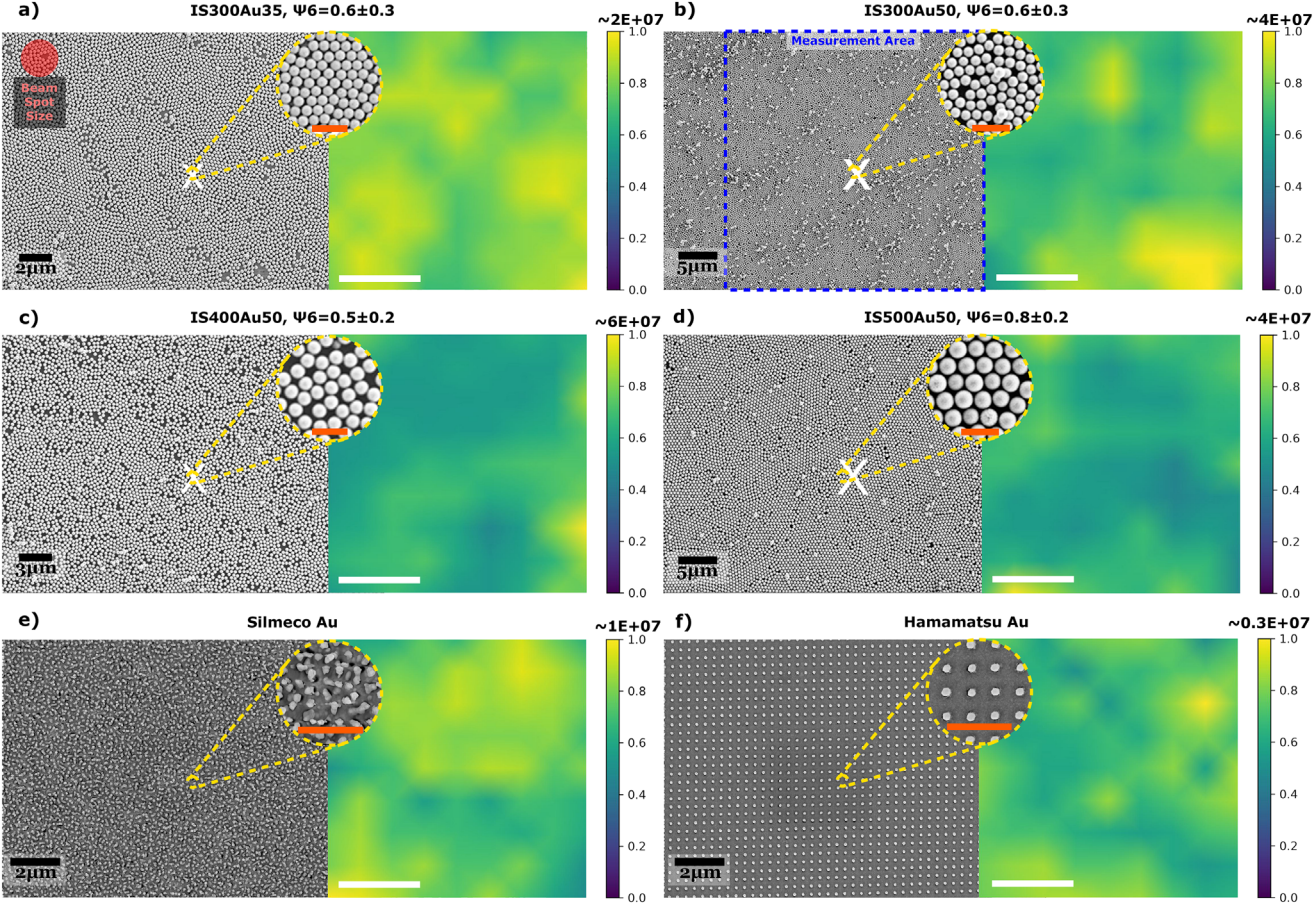
SEM image and corresponding EF distribution maps for the 1608 cm^−1^ peak of BPE of the coordinate‐translated areas of the following substrates: IS300Au35 (a), IS300Au50 (b), IS400Au50 (c), IS500Au50 (d), Silmeco Au (e), and Hamamatsu Au (f). These heatmaps are max‐normalized to make the distributions comparable. The blue dashed rectangle indicates the size of the measured area, while the red circle represents the size of the beam spot. All results are based on a 1 µM BPE deposition. The white scale bars represent 10 µM, while the orange ones in the insets represent 1 µm. The corresponding bond order parameter $\Psi_{6}$ is determined for the respective colloidal monolayers in (a)–(d).

As elaborated in Section 2.1, the colloids on the fluorocarbon‐coated silicon substrate show some variation in their structural order. These include a close‐packed monolayer with some additional particles sparsely distributed on top (Figure 4a,b), a highly ordered monolayer (Figure 4d), or a monolayer with defects (Figure 4c). We quantify the degree of order of the monolayers by means of the Voronoi tesselation approach and their corresponding bond order parameter $\Psi_{6}$ discussed in Section 2.1 is shown in respective Figures 4a–d. This value accounts for vacancies, grain boundaries, and secondary particles scattered on the monolayer. We selected up to eight locations per sample representing these different distributions using SEM imaging and used coordinate translation to retrieve them under a Raman microscope.

A subset of the top‐performing substrates from the particle size and coating thickness study (Section S1) was selected for SERS analysis. Table S3 summarizes the results of this study.

To avoid confusion between the two experimental datasets, Table 1 summarizes the EF values obtained without coordinate translation and those obtained with coordinate translation for the same representative substrates, comparing them to values achieved via electromagnetic simulation. The max‐normalized EF distributions for this subset, which have the highest mean EF among the selected points, are shown in Figure 4. Analyzing the normalized EF distribution reveals how uniformly the enhancement is distributed across the measured area. Most EF values for all substrates fall within the yellow‐to‐green range in the top half of the color bars. This shows that the majority of them are relatively high, clustering around mid‐to‐high values. Compared to the commercial substrates, the fabricated substrates' EF values show a lower spatial signal inhomogeneity in the maps. Additionally, as these maps are obtained from coordinate‐translated measurements that link structure and response [40], they enable a direct assessment of how local structural order correlates with the SERS signal statistics, considering the limits of the measurement techniques. Overall, signal inhomogeneity may decrease with an increasing degree of structural order, as can be seen in Figure 4d, which shows a SERS CV of ∼20% at $\Psi_{6}=0.8$, while Figure S3 shows a CV of ∼28% at $\Psi_{6}=0.7$. This trend is consistent with defect engineering: coating‐dependent changes in interparticle spacing and nanogap geometry modulate light localization. Intentional defects have been used to boost SERS; for example, Ananthoju et al. introduced vacancy defects in graphene that serve as anchoring sites for Au nanoparticles, thereby controlling metal–metal nanogaps that host SERS hot spots [41]. As seen in the simulations (Figure 2), which resemble the close‐packed structure of Figure 4d, the coating can somewhat fill the gap between the structures. When the distance between the particles is increased, e.g., Figure 4c (no grains identified, indicating low close‐packed structural order, even locally) compared to Figure 4d (22 grains identified, highlighting close‐packed structural order as 46% of particles are part of an identified grain), the volume and magnitude of the localized electromagnetic field, i.e., hotspot, increase. The same reasoning applies to monolayers with additional particles scarcely distributed on top (defect), which leads to a relative low $\Psi_{6}$‐value and only 2.1% of the 300 nm colloids that are part of one of the nine identified grains, as shown in Figure 4b. Bauman et al.'s study, which used ligands to tune sub‐nanometer gaps between monolayer particles, further supports this reasoning [42]. Green et al. reached a similar conclusion when studying the near‐field finite element simulations [25].

**Table 1:** Overview of the mean and maximum experimental (Exp.) enhancement factor (EF) values, calculated from the BPE peak at 1608 ${\mathrm{cm}}^{-1}$ for the coordinate‐translated points on selected SERS substrates, compared with the idealized simulation (Sim.) of a doublet on a gold‐coated surface. The top experimental results are boxed.

SERS substrate	Exp. Mean EF	Exp. Max. EF	CT^*^ Exp. Mean EF	CT Exp. Max. EF	Sim. Mean EF	Sim. Max. EF
IS300Au35	3E+07	6E+07	1E+07	2E+07	3E+00	2E+02
IS300Au50	4E+07	6E+07	2E+06	3E+07	3E+00	4E+01
IS400Au50	—	—	3E+07	$\begin{array}{c} 5E+07 \end{array}$	1E+05	1E+08
IS500Au50	9E+07	$\begin{array}{c} 1E+08 \end{array}$	3E+07	$\begin{array}{c} 5E+07 \end{array}$	1E+01	2E+04

^*^ CT: measurements with coordinate translation.

*Note*: Experimental maximum and mean values were computed over a 9 × 9 measurement grid across a 30 × 30 µm^2^ area, whereas simulated maximum and mean values were calculated within a 0.1 µm × 1 µm simulation window (magenta dotted rectangle in Figure 2); therefore, these metrics are not directly comparable on a one‐to‐one basis.

To exclude any colloidal size and Au‐thickness effect when making a fair comparison between the structural order of the assembled monolayers and their corresponding SERS performance, we selected another area of the IS500Au50 substrate, as shown in Figure S3 (cf. ESI). From these two distinct areas, it can be inferred that the more uniformly distributed close‐packed monolayer in Figure 4d (($\Psi_{6}=0.8\pm 0.2)$ shows a more homogeneous EF distribution (CV≈20%) compared to the slightly less‐ordered monolayer in Figure S3a ($\Psi_{6}=0.7\pm 0.3)$ with a CV of ≈28% in EF‐values. This is attributed to the long‐range order in Figure 4d having 22 grains with 42% of the colloids, while Figure S3 has more grains, but only 27% of the particles are part of the 28 identified grains. The latter elucidates the presence of local ordering with many grain boundaries and vacancies. Consequently, this results in an inhomogeneous SERS signal distribution. The more uniformly close‐packed monolayer in Figure 4d has more secondary particles on top. We suspect that the more uniform order and these secondary particles leads to higher maximum EFs compared to Figure S3 with much more grain boundaries and defects.

**Figure 5:**
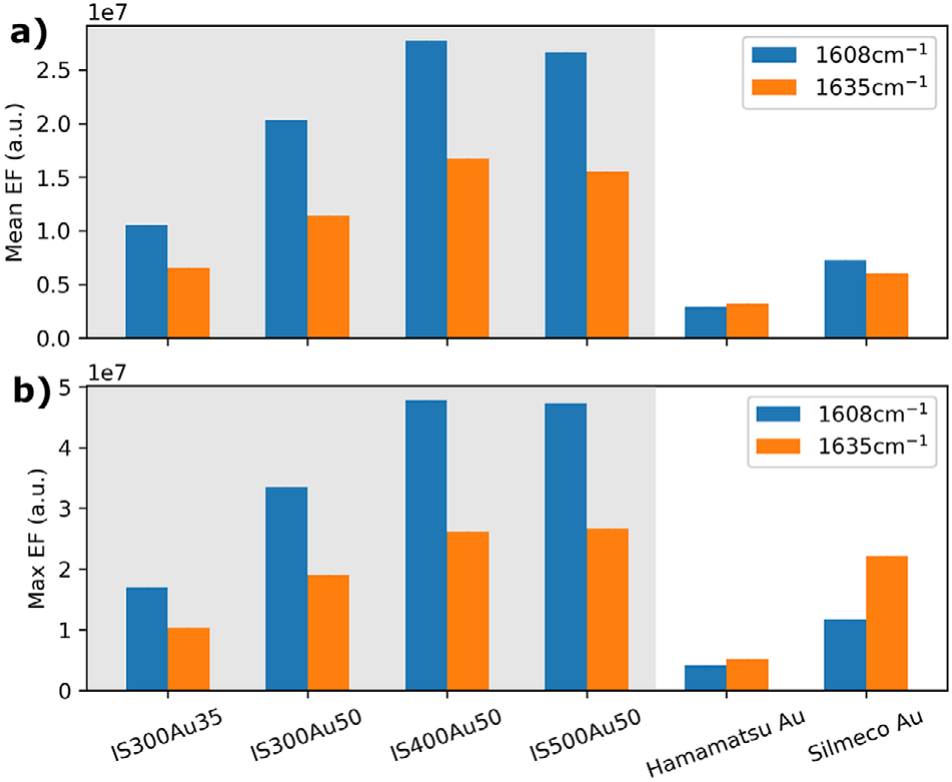
SERS performance metrics for the coordinate‐translated areas of substrates with optimum coating, including the mean enhancement factor (a) and maximum enhancement factor (b). These values are averaged across multiple measured areas, with all results based on a 1 µM BPE deposition.

**Figure 6:**
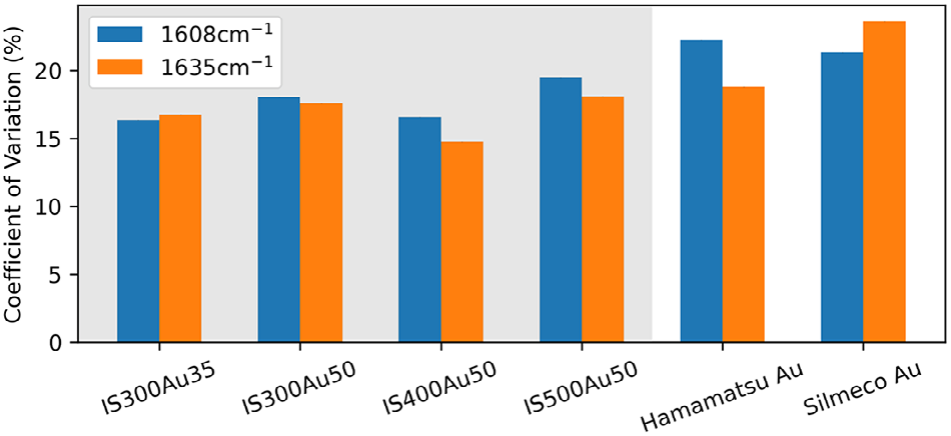
Coefficient of variation over area for the coordinate‐translated areas of substrates with optimum coating. These values are averaged across multiple measured areas, with all results based on a 1 µM BPE deposition.

The summary of the substrates' performance in terms of EF and spatial signal inhomogeneity averaged over all measured points is given in Figures 5 and 6 and Table S3. Compared to the visually selected points summarized in Figure S2, a similar trend is observed regarding particle size and coating thickness. The optimum particle size is 400–500 nm, which agrees with the previous results. The optimum coating was previously found to be around 35–50 nm for the 300 nm particles. This is further clarified in Figure 5, which shows an improved performance for the selected points in the case of 50 nm coating. The top‐performing substrates are IS400Au50 and IS500Au50, with a maximum EF of 5 × 10^7^ and a mean EF of 3 × 10^7^ for the 1608 cm^−^ BPE peak. Achieving the best performance for particles in the 400–500 nm range with a 50 nm gold coating aligns with prior studies on particle size and coating thickness reported in the literature [43].

For reference, Table 1 juxtaposes the experimental mean and maximum EF values of these representative substrates with the corresponding simulated electromagnetic enhancement factors. These numerical differences should be interpreted cautiously, because the experimental statistics are computed over a microscope map, whereas the simulated statistics are extracted from an idealized sub‐micron window, and thus they do not represent a one‐to‐one matched sampling.

Within the simulated sweep in Table S1, the highest simulated mean and maximum occur for IS400Au35 (Sim. Mean EF =2E+08; Sim. Max EF =4E+12), whereas IS500Au50 yields Sim. Mean EF =1E+01 and Sim. Max EF =2E+04, corresponding to 7E−08 × (mean) and 5E−09 × (max) of the sweep optimum; for a size‐matched comparison within the 500 nm series, the simulated mean is highest at IS500Au35 (Sim. Mean EF =3E+01), making IS500Au50 0.44 × (56% lower) than the mean‐optimum, while it coincides with the maximum‐optimum in the 500 nm series (Sim. Max EF =2E+04).

The area‐to‐area CV, i.e., spatial signal heterogeneity, of the IS300Au35 and IS500Au65 substrates, was found to be higher than that of commercial substrates in FiAmong coordinate‐translated points, IS300Au35 has the lowest CV, 16%, among the measured substrates. The top‐performing substrate, IS400Au50, has a CV of 17%, making it the second most stable. While some studies report coefficients of variation as low as 3% or less [27], SERS signal variations can be influenced by specific measurement conditions, such as the analyte and signal‐to‐noise ratio [40]. A substrate‐wide heatmap of the 1608 cm^−1^ peak's intensity is presented in Figure S4.

**Figure 7:**
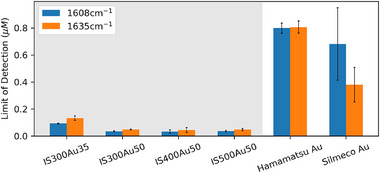
Limit of detection for the coordinate‐translated areas of substrates with optimum coating. These values are averaged across multiple measured areas. The error bars show the area‐to‐area variation.

Regarding sensitivity, all fabricated substrates can achieve LODs in the tens of nM range, which is roughly at least ten times more sensitive than commercial substrates, as can be seen in Figure 7 and Table S3. The top‐performing substrate, IS400Au50, provides a significantly higher sensitivity than Hamamatsu Au and Silmeco Au, with ian mprovement of around 24‐fold and 21‐fold, respectively. The superior SERS signal of the fabricated substrates is further highlighted in the error bars of Figure 7, depicting the area‐to‐area variation in LOD. This originates from these substrates' higher signal‐to‐noise ratio as well as their lower spatial signal inhomogeneity. The evolution of the 1608 cm^−1^ peak intensity of BPE with increasing concentration is given in Figure S5; in this figure, the sigmoid fit is used as a guidance tool to objectively identify the approximately linear working range of the experimental response.

Altogether, these results confirm that enhanced SERS performance can be achieved using affordable substrates, as we can create controlled close‐packed monolayers with a good quality structural order on a substrate of 20 × 20 mm^2^.

## 3 Conclusion

Our work demonstrates that exploiting triboelectrification‐driven self‐assembly can rapidly produce cost‐effective and reproducible substrates covered with close‐packed colloidal monolayers, suitable for use as high‐performance SERS substrates. We identified key parameters influencing signal enhancement and reproducibility by systematically varying particle size and gold coating thickness. The triboelectric‐driven assembled monolayer SERS substrates comprising monodisperse spherical colloids of 300, 400, and 500 nm resulted in maximum EF‐values of > × 10^7^ both with and without coordinate translation. In the particle size and coating thickness study without coordinate translation, the 500 nm particles with a 50 nm gold coating (IS500Au50) produced the highest maximum EF of 1 × 10^8^ (9% CV), surpassing commercial substrates from Silmeco by one order of magnitude and Hamamatsu by two orders of magnitude. The LOD of this substrate for BPE was 36 nM. Substrates with 400 nm particles and a 50 nm gold coating had a maximum EF of 5 × 10^7^, LOD of 33 nM, and a CV of 17%. These findings highlight the effectiveness of triboelectrification in efficiently and cost‐effectively producing homogeneous monolayers that can surpass commercial SERS substrates in performance and reproducibility, thereby addressing the limitations of traditional fabrication methods, such as the more complex assembly alternatives, like wet chemical assembly, or the significantly more costly ion beam lithography. The success of this approach positions triboelectrification‐driven self‐assembly as a promising contender for large‐scale SERS substrate production, with broad applications in biosensing, diagnostics, and trace chemical detection, enabling the widespread adoption of SERS in various scientific and industrial applications.

## 4 Experimental

### 4.1 Lumerical Simulations

For the optical simulation of the nanostructures, we used Lumerical software (Ansys), specifically the Finite‐Difference Time Domain (FDTD) solver [44]. We conducted around 250 2D simulations. These simulations varied particle size from 100 to 800 nm (with a step size of 50 nm) and coating thicknesses from 10 to 85 nm (with a step size of 5 nm), using Lumerical's Python API. The material for the substrate was “Si (Silicon) ‐ Palik”, particles “SiO2 (Glass) ‐ Palik”, and the coating “Au (Gold) ‐ Johnson and Christy”. A planar source with a wavelength of 785 nm was used for the simulations; this wavelength was commonly used in biosensing applications, which was the ultimate target of this development. The boundary conditions were set to a perfectly matched layer (PML) for top and bottom boundaries and periodic for the sides. The core‐shell structure was simulated using the mesh accuracy option when defining the geometries. Finally, we employed two discrete Fourier transform (DFT) monitors one covering the entire simulation area and another covering a 0.1 × 1 µm^2^ rectangle between the particles. The positions of the source and monitors remained constant for each configuration in the sweep. To avoid grid‐quantization (“staircasing”) artifacts that could spuriously elevate window‐averaged fields, we screened the sweep and excluded affected instances; experimentally irrelevant parameter combinations (e.g., coating thickness ≈ particle size) were also omitted and shown as blank cells. To report a SERS‐relevant metric from the simulated near‐fields, we computed an electromagnetic enhancement factor using the standard approximation $\mathrm{EF}\approx {\left|E/E_{0}\right|}^{4}$ at the excitation wavelength and summarized it by its spatial mean and maximum within the fixed analysis window (magenta dotted rectangle in Figure 2) [45].

### 4.2 Fabrication of the SERS Substrates

Substrates are produced using the rubbing assembly technique, described elaborately in ref. [35]. Unless otherwise stated, PDMS stamps were used. The PDMS (10:1 w/w) stamps were made by pouring PDMS (SYLGARD 184 silicone elastomer kit; Dow, Inc.) into a Petri dish and cross‐linking it in an oven at 65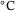 for at least 4 h. The PDMS was eventually cut into pieces of 1 × 1 cm^2^.

### 4.3 SERS Performance Analyte

1,2‐bis‐(4‐pyridyl) ethylene (BPE) from Sigma–Aldrich was used for SERS measurements. To prepare the stock solution, 36.4 mg of BPE powder (molecular weight 182.22 g mol^−1^) is dissolved in 20 mL of 99.7% pure ethanol, resulting in a 10 mM solution. 0.2–1 µM (0.2 µM step) and 1–5 µM (1 µM step) concentrations were prepared by diluting the stock solution with ethanol for SERS characterization.

### 4.4 Scanning Electron Microscopy

Scanning electron microscopy (SEM) was carried out using a Merlin high‐resolution scanning electron microscope (ZEISS) with an accelerating voltage of 1.4 kV with a high efficiency secondary electrons (HE‐SE2) detector.

### 4.5 Raman and SERS Measurements

For the Raman measurements, we used an InVia spectrometer (Renishaw) paired with a DM2700 optical bright‐field microscope (Leica). A 785 nm excitation wavelength from a diode‐pumped solid‐state laser (Renishaw) was chosen, as it was commonly used in biosensing applications, which was the ultimate target of this development. The sample was illuminated using a Leica N PLAN 20X/0.4 objective, delivering 234 µW of power over a 2.5 µm diameter beam spot. The exposure time for the Raman and SERS measurements were set to 50 and 0.5 s, respectively. The collected Raman signal was directed onto a Centrus CCD detector (Renishaw) through a 1200 lines/mm grating. We focused on the spectral range of 614–1720 ${\mathrm{cm}}^{-1}$ with a resolution of 1 ${\mathrm{cm}}^{-1}$, targeting the most prominent peaks of BPE. To determine the enhancement factor (EF), spontaneous Raman measurements were done in 20 µL droplets placed on 50‐nm metal‐coated coverslips (AU.0500, Platypus Technologies). The measurement settings were the same as SERS but with a 50 s acquisition time, instead of 0.5 s, chosen based on the evaporation rate of ethanol.

### 4.6 Data Acquisition and Analysis

A 44.5 µL aliquot of BPE‐ethanol solution was drop‐cast onto a 4 cm^2^ substrate area for SERS characterization [45, 46]. Per substrate, multiple 15 µm × 15 µm maps with 2 µm steps (9 × 9 spectra) were measured to investigate the point‐to‐point homogeneity of the performance. These maps were retrieved where appropriate based on coordinate‐translated microscopy between a SEM and Raman microscope [40]. The spectra's baselines were subtracted using Asymmetric Least Squares (ALS) [47], ensuring that the intensity variations did not come from the fluorescent background. The standard deviation (STD), EF, and LOD were calculated for the two strongest characteristic peaks of the analyte individually. The CV over space was calculated over 81 spectra measured in the maps. The most widely used definition of the SERS EF [48], shown in Equation (1), was used here.

(1) $$\text{EF}=\frac{I_{\text{SERS}}/N_{\text{SERS}}}{I_{\text{NR}}/N_{\text{NR}}}$$

To elaborate, $I_{\text{SERS}}$ is the intensity of the SERS peak, and $N_{\text{SERS}}$ is the number of molecules on the surface contributing to the SERS signal. Similarly, $I_{\text{NR}}$ denotes the intensity of the same peak in the spontaneous Raman spectrum, while $N_{\text{NR}}$ corresponds to the number of molecules in the laser's collection volume during the spontaneous Raman measurement.

The beam spot area, calculated using the Airy disk formula, was used to estimate $N_{\text{SERS}}$ assuming a uniform molecular distribution across the surface. However, the same justification did not apply to the coffee rings on a nonstructured surface. Thus, $I_{\text{NR}}$ was measured in a homogeneous solution. The main contribution to $I_{\text{NR}}$ comes from the homogeneously distributed molecules in the collection volume, calculated as the area of the beam spot multiplied by the confocality height. The confocal height was estimated as the full width at half maximum (FWHM) of the 520 cm^−1^ Raman peak of a silicon target obtained from a height scan and was 87 μm. Equation (2) was used to calculate LOD based on the STD of the signal without analyte (σ) and the slope of the calibration curve (S) [49]. σ was taken to be the time‐STD of the peak intensity without any analyte deposited on the substrate. The slope S was obtained from a linear regression on the subset of calibration points within the approximately linear working range of the concentration–intensity response. This linear range was identified using a sigmoid fit to the full concentration sweep as an objective selection aid [40]. Furthermore, the limit of quantification (LOQ) has the same formula with a factor of 10 instead of 3.3.

(2) $$\text{LOD}=\frac{3.3\sigma }{S}$$

## Conflicts of Interest

The authors declare no conflicts of interest.

## Supporting information


**Supporting File**: smtd70570‐sup‐0001‐SuppMat.pdf

## Data Availability

The data that support the findings of this study are available from the corresponding author upon reasonable request.
