# Supplementary material for: SERS Substrate Fabrication via Rapid Triboelectrification‐Driven Self‐Assembly of Close‐Packed Colloidal Monolayers
Source: Small Methods. 2026 Mar 1;10(6):e01660. doi: 10.1002/smtd.202501660 (PMC13010213; doi:10.1002/smtd.202501660)
Supplement: Supplementary file 1 — Supporting File: smtd70570‐sup‐0001‐SuppMat.pdf [file SMTD-10-e01660-s001.pdf]

Electronic Supplementary Information

SERS Substrate Fabrication via Rapid Triboelectrification-Driven Self-Assembly of Close-Packed Colloidal Monolayers

Mehdi Feizpour[1] (🖂), Ignaas Jimidar (🖂)[2,3], Gert Desmet[3], Heidi Ottevaere[4]

[1] *Vrije Universiteit Brussel, Department of Applied Physics and Photonics, Brussels Photonics, Pleinlaan 2, 1050 Brussel, Belgium*
[2] *University of Twente, Mesoscale Chemical Systems, MESA+ Institute for Nanotechnology and Faculty of Science and Technology, 7500 AE Enschede, The Netherlands*
[3] *Vrije Universiteit Brussel, Department of Chemical Engineering, Pleinlaan 2, 1050 Brussel, Belgium*
[4] *Vrije Universiteit Brussel and Flanders Make, Department of Applied Physics and Photonics, Brussels Photonics, Pleinlaan 2, 1050 Brussel, Belgium*

** Address correspondence to Mehdi Feizpour, mehdi.feizpour@vub.be; Ignaas S.M. Jimidar, i.s.m.jimidar@utwente.nl*

S1 Effect of Coating Thickness and Particle Size on SERS Performance

To investigate the effect of the gold thickness on the SERS performance, substrates prepared with monolayers of 300 and 500 nm particles were gold-coated with 20, 35, 50 and 65 nm thicknesses. Multiple homogeneous areas, selected by visual inspection of the diffracted colors, were mapped over these substrates. Fig. S2 and Table S summarize the mean EF (Fig. S2a), maximum EF (Fig. S2b), and the spatial coefficient of variation (CV) in signal (Fig. S2c) averaged over the 15 µm × 15 µm maps measured maps.

For both particle sizes, EF increases as the coating thickness grows, reaches a peak, and then decreases, highlighting that an optimal thickness should be found for an optimal SERS performance. This agrees with the trade-off highlighted in Fig. **Error! Reference source not found.**. The optimal coating thickness is 35-50 nm for the substrates with 300 nm particles and 50 nm for the substrates with 500 nm particles. The slight difference between these optimal values and the simulations' results can be attributed to the idealized conditions, as noted in Section 2.2.

Across all coating thicknesses, the substrates with 300 nm particles generally perform worse than the substrates with 500 nm particles, except for the maximum EF at a coating thickness of 35 nm. This might be an artifact of the visual selection of the investigated points and requires further validation. The sample IS500Au50 (500 nm particles with a 50 nm gold coating) shows the highest EF of 1×10^{8} for the 1605 cm^{-1} peak of BPE. This represents a 10-fold improvement over Silmeco Au substrates and nearly a ~100-fold increase compared to Hamamatsu Au substrates. The SERS spectra for IS500Au50 compared to commercial SERS substrates from Hamamatsu and Silmeco are presented in Fig. **Error! Reference source not found.**. IS500Au50's 1605 cm-1 peak is ~6 times stronger than Silmeco Au (nanograss) and ~19 times stronger than Hamamatsu Au (nanopillars) under similar conditions and using the same measurement settings.

The area-to-area CV in Fig. S2c and Table S highlights the spatial instability of the different substrates, primarily caused by defects and inhomogeneities in the self-assembled structure. Fig. S2c shows higher instability for substrates with 300 nm particles compared to the substrates with 500 nm particles, which is expected as it is more challenging to assemble the 300 nm particles than the 500 nm ones, as the smaller ones tend to have stronger cohesive interactions and concomitant larger aggregates, that inhibits their assembly into close-packed monolayers over large areas. Nevertheless, factors like defects in metal coating, such as incomplete coverage, variations in hotspot strength due to irregular particle arrangements or gaps between particles and uneven analyte distribution across the substrate can also contribute to the observed variations in signal [40]. The IS500Au50 sample (500 nm particles with 50 nm gold) shows the lowest instability at 9%. Instability decreases with increasing coating thickness up to 50 nm, but rises beyond that, likely due to reduced enhancement (Fig. S2a) and unchanged structural defects.

Since the measurement locations on the substrate were selected visually in this part of the study, unexpected structural variations in the assembly may have influenced the results. Thus, SEM-to-Raman microscope coordinate-translated measurements can be used to confirm these findings and account for any structural differences (cf. section 2.4).

Table S1. Simulated electromagnetic enhancement factor statistics (EF) for the 300 – 500 nm particle-size range and 35 – 65 nm Au coating thickness range. Simulated values were computed from the near-field using the E^4 approximation, EFEM ≈ $|E/E0|^4$, and summarized by the spatial mean, maximum, and standard deviation within the fixed 0.1 μm × 1 μm analysis window (magenta dotted rectangle in Fig. 2).

SERS Substrate	Sim. Mean EF	Sim. Max EF	Sim. Std EF
IS300Au35	3E+00	2E+02	5E+00
IS300Au40	3E+00	3E+01	5E+00
IS300Au45	3E+00	3E+01	5E+00
IS300Au50	3E+00	4E+01	5E+00
IS300Au55	3E+00	2E+01	5E+00
IS300Au60	7E+07	2E+11	2E+09
IS300Au65	1E+07	3E+10	3E+08
IS350Au35	2E+06	9E+09	8E+07
IS350Au40	2E+06	3E+10	2E+08
IS350Au45	2E+06	5E+10	2E+08
IS350Au50	3E+05	4E+08	2E+06
IS350Au55	1E+05	7E+07	4E+05
IS350Au60	1E+05	3E+07	5E+05
IS350Au65	5E+04	4E+06	2E+05
IS400Au35	2E+08	4E+12	3E+09
IS400Au40	2E+05	5E+08	2E+06
IS400Au45	3E+03	2E+05	5E+04
IS400Au50	1E+05	1E+08	2E+06
IS400Au55	3E+02	7E+04	1E+04
IS400Au60	2E+02	1E+04	4E+03
IS400Au65	1E+02	5E+03	2E+03
IS450Au35	5E+07	1E+11	4E+08
IS450Au40	4E+07	2E+11	6E+08
IS450Au45	2E+07	7E+10	5E+08
IS450Au50	2E+04	2E+06	2E+05
IS450Au55	5E+03	2E+05	4E+04
IS450Au60	4E+03	2E+05	4E+04
IS450Au65	2E+03	2E+04	2E+04
IS500Au35	3E+01	2E+04	1E+02
IS500Au40	2E+01	1E+04	8E+01
IS500Au45	2E+01	2E+03	4E+01
IS500Au50	1E+01	2E+04	6E+01
IS500Au55	1E+01	8E+02	2E+01
IS500Au60	9E+00	3E+03	2E+01
IS500Au65	8E+00	5E+02	1E+01

Table S2. An overview of the mean and maximum EF values and CV calculated for the 1608 cm^{-1} peak of BPE.

SERS Substrate	Mean EF (a.u.)	Maximum EF (a.u.)	Coefficient of Variation (%)
**IS300Au20†**	7E+06	1E+07	11%
**IS300Au35**	3E+07	6E+07	34%
**IS300Au50**	4E+07	6E+07	19%
**IS300Au65**	2E+07	3E+07	15%
**IS500Au20**	6E+06	8E+06	16%
**IS500Au35**	3E+07	5E+07	10%
**IS500Au50**	9E+07	1E+08	9%
**IS500Au65**	5E+07	1E+08	29%
**Hamamatsu Au**	3E+06	4E+06	22%
**Silmeco Au**	7E+06	1E+07	21%

* STD: standard deviation over a 15 μm × 15 μm map

† IS300Au20: Substrate with 300 nm particles and 20 nm Au coating

‡ ISP500Au100: Substrate with 500 nm particles and 100 nm Au coating

Table S3. An overview of the mean and maximum EF values and CV calculated for the 1608 cm^{-1} peak of BPE for the coordinate-translated points.

SERS Substrate	Mean EF (a.u.)	Maximum EF (a.u.)	LOD (nM)	Coefficient of Variation (%)
**IS300Au35†**	1E+07	2E+07	93	16%
**IS300Au50**	2E+06	3E+07	35	18%
**IS400Au50**	3E+07	5E+07	33	17%
**IS500Au50**	3E+07	5E+07	36	19%
**Hamamatsu Au**	3E+06	4E+06	800	22%
**Silmeco Au**	7E+06	1E+07	680	21%

* STD: standard deviation over a 15 μm × 15 μm map

† IS300Au50: Substrate with 300 nm particles and 50 nm Au coating

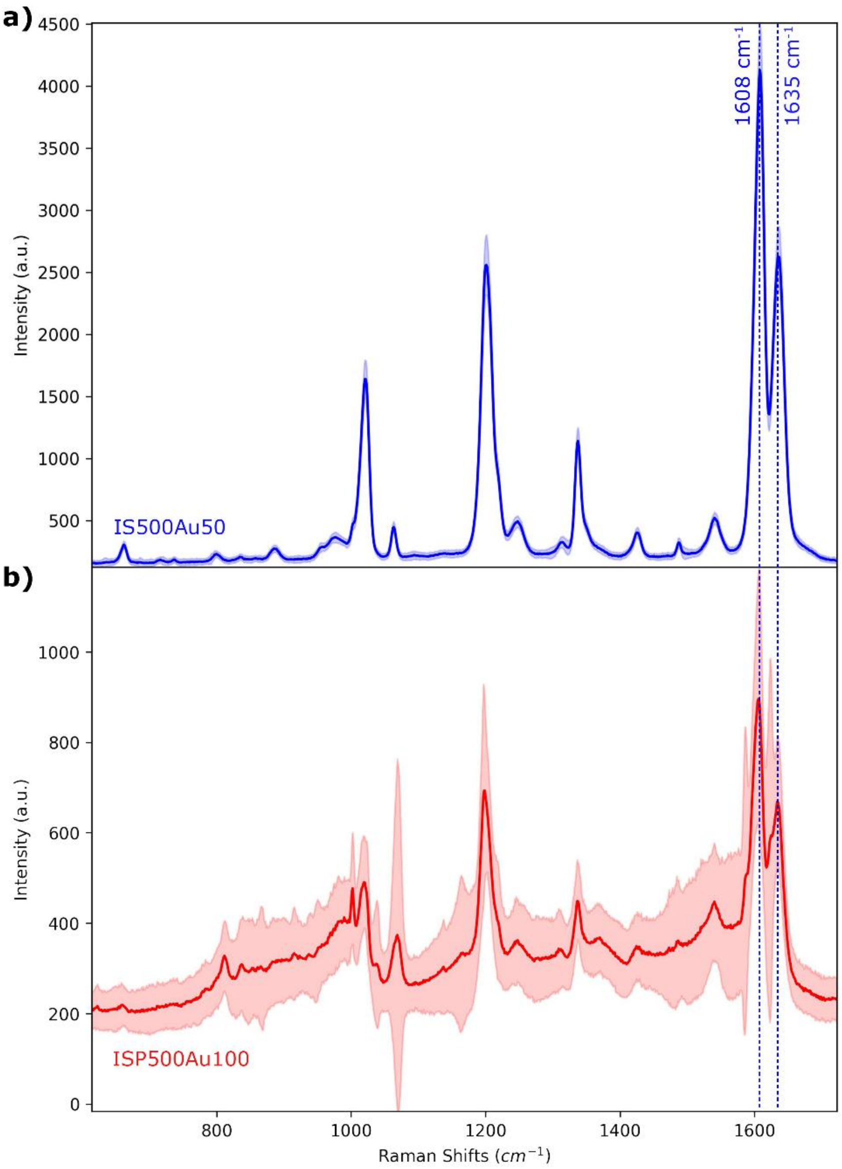

Figure S1. Mean SERS spectra of a 1 μM BPE deposition on IS500Au50 (a) and ISP500Au100 (b) with their spatial standard deviation. IS500Au50 corresponds to 500 nm particles with 50 nm of gold coating, and ISP500Au100 corresponds to 500 nm particles with 100 nm of gold coating.

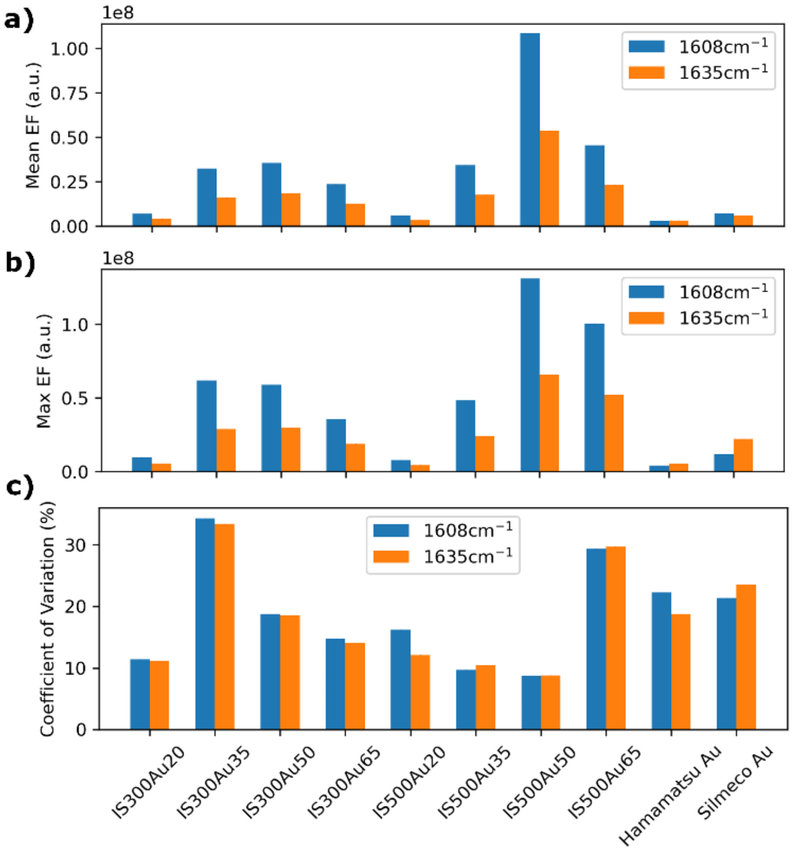

Figure S2. SERS performance metrics, including the mean enhancement factor (a), maximum enhancement factor (b) and the coefficient of variation (CV) in peak intensity (c), averaged across multiple measured areas for various combinations of particle sizes and coating thicknesses. All results are based on a 1 μM BPE deposition.

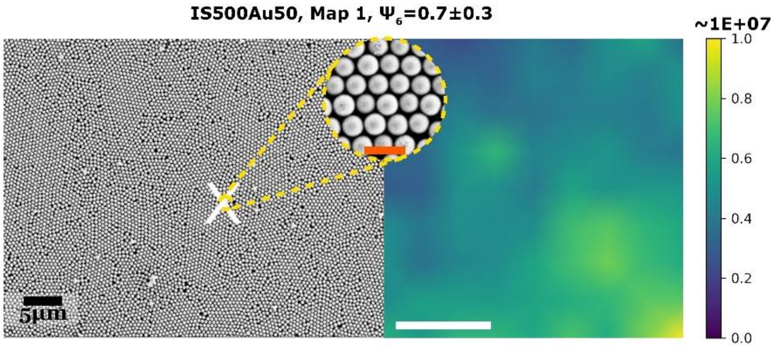

Figure S3. A SEM image (left side) and corresponding EF distribution map (right side) at the 1608 cm^{-1} BPE band for a coordinate-translated measurement area on an IS500Au50 substrate brought in comparison to Fig. 4d. The corresponding bond order value Ψ_6 is shown above the panel. The EF heatmap is max-normalized (0–1) to enable comparison; the approximate absolute EF maximum is indicated above the color bar. The inset shows a higher-magnification view of the particle packing. The black scale bar in the SEM image represents 5 μm; the white scale bar in the EF map represents 10 μm; the orange scale bar in the inset represents 1 μm.

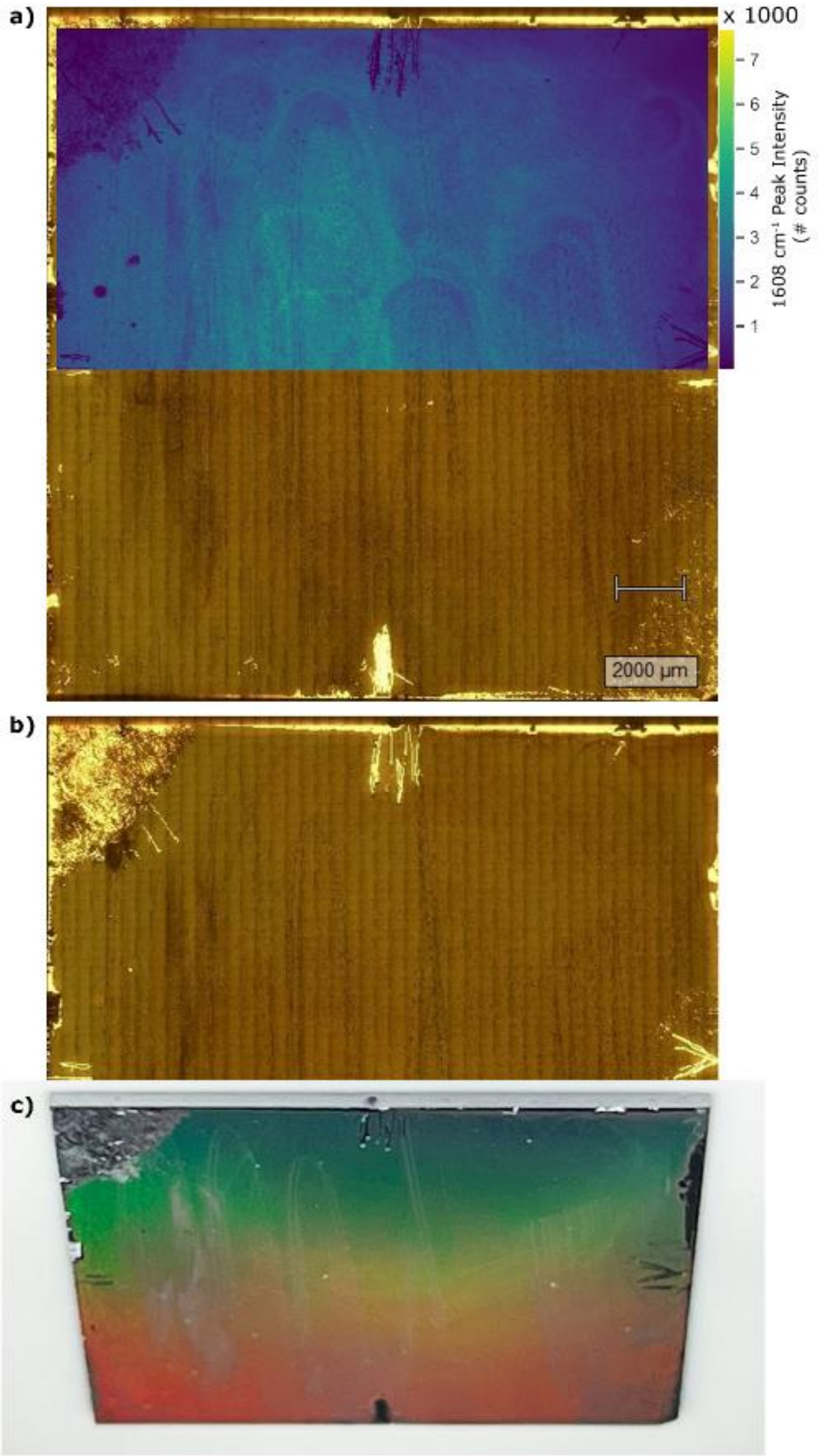

Figure S4. The 1608 cm-1 baseline-corrected peak intensity heatmap overlayed on the white light microscopy image of IS500Au50 for 19 µM deposited BPE (a). The top part of the substrate that is covered in subfigure a (b). A photograph of the substrate highlighting diffracted white light (c).

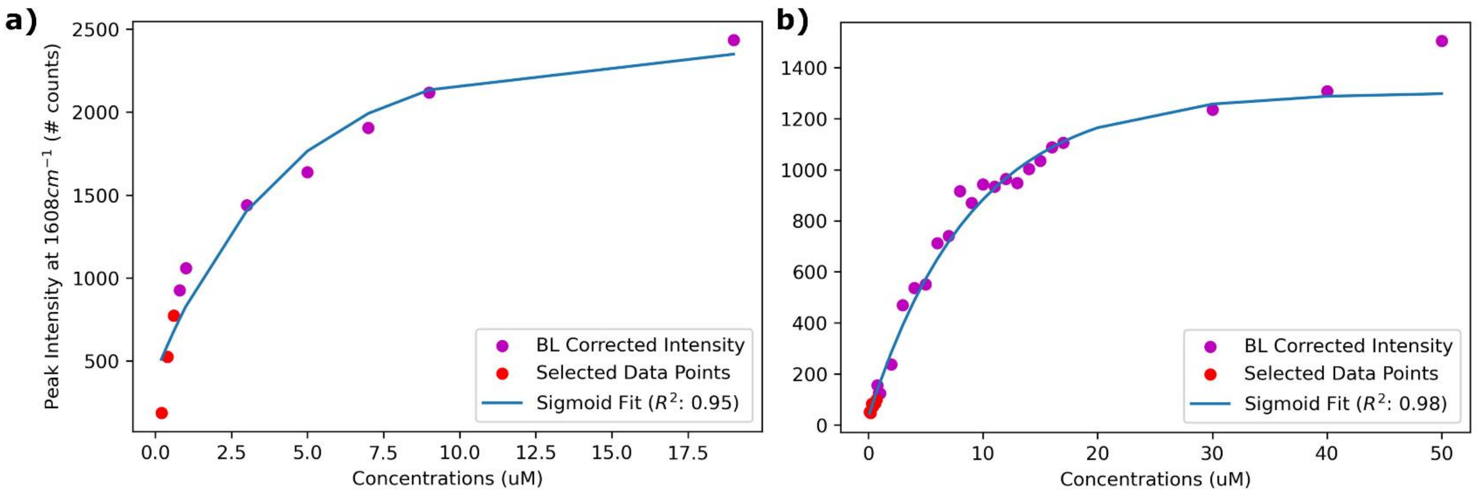

Figure S5. The 1608 cm-1 peak intensity versus BPE concentration i.e. calibration plot for IS400Au50 (a) and Hamamatsu Au (b). Magenta markers show the baseline-corrected experimental intensities, and the blue curve is a sigmoid fit used only to identify the approximately linear working range within the full extended-range response. Red markers indicate the subset of experimental points selected from this linear range and used for the subsequent linear regression that defines the calibration used for the LOD calculation.

Abbreviations

EF: enhancement factor

SERS: Surface-enhanced Raman spectroscopy

LOQ: limit of quantification

LOD: limit of detection

STD: standard deviation

CV: coefficient of variation

BPE: trans-1,2-bis(4-pyridyl)ethylene

SEM: scanning electron microscope

IS300Au20: SERS substrate - 300 nm particles with 20 nm Au

IS300Au35: SERS substrate - 300 nm particles with 35 nm Au

IS300Au50: SERS substrate - 300 nm particles with 50 nm Au

IS300Au65: SERS substrate - 300 nm particles with 60 nm Au

IS400Au20: SERS substrate - 400 nm particles with 20 nm Au

IS400Au35: SERS substrate - 400 nm particles with 35 nm Au

IS400Au50: SERS substrate - 400 nm particles with 50 nm Au

IS400Au65: SERS substrate - 400 nm particles with 60 nm Au

IS500Au20: SERS substrate - 500 nm particles with 20 nm Au

IS500Au35: SERS substrate - 500 nm particles with 35 nm Au

IS500Au50: SERS substrate - 500 nm particles with 50 nm Au

IS500Au65: SERS substrate - 500 nm particles with 60 nm Au
